# Advances of Salivary Proteomics in Oral Squamous Cell Carcinoma (OSCC) Detection: An Update

**DOI:** 10.3390/proteomes4040041

**Published:** 2016-12-15

**Authors:** Rabia Sannam Khan, Zohaib Khurshid, Shazia Akhbar, Syed Faraz Moin

**Affiliations:** 1 Department of Oral Pathology, College of Dentistry, Baqai University, Super Highway, P.O. Box: 2407, Karachi 74600, Pakistan; rabia.sannam.khan@gmail.com; 2 Department of Prosthodontics and Implantology, College of Dentistry, King Faisal University, Al-Ahsa 31982, Saudi Arabia; 3 Department of Oral Pathology, Dow Dental College, Dow University of Heath Sciences (DUHS), Baba-E-Urdu Road, Karachi 74200, Pakistan; shazia.akhbar@duhs.edu.pk; 4 National Center for Proteomics, University of Karachi, University Road, Karachi 75270, Pakistan; faraz.moin@uok.edu.pk

**Keywords:** saliva, biomarkers, oral squamous cell carcinoma, diagnosis

## Abstract

Oral cancer refers to malignancies that have higher morbidity and mortality rates due to the late stage diagnosis and no early detection of a reliable diagnostic marker, while oral squamous cell carcinoma (OSCC) is amongst the world’s top ten most common cancers. Diagnosis of cancer requires highly sensitive and specific diagnostic tools which can support untraceable hidden sites of OSCC, yet to be unleashed, for which plenty of biomarkers are identified; the most recommended biomarker detection medium for OSCC includes biological fluids, such as blood and saliva. Saliva holds a promising future in the search for new clinical biomarkers that are easily accessible, less complex, accurate, and cost effective as well as being a non-invasive technique to follow, by analysing the malignant cells’ molecular pathology obtained from saliva through proteomic, genomic and transcriptomic approaches. However, protein biomarkers provide an immense potential for developing novel marker-based assays for oral cancer, hence this current review offers an overall focus on the discovery of a panel of candidates as salivary protein biomarkers, as well as the proteomic tools used for their identification and their significance in early oral cancer detection.

## 1. Introduction

Oral squamous cell carcinoma (OSCC) is the sixth most common malignancy worldwide [1]. The development of OSCC occurs due to alteration in gene expression affected by specific genetic characteristics and environmental conditions, including tobacco, betel quid, alcoholic beverages, chronic inflammation and viral infections [2]. In spite of breakthroughs in recognition and management, no significant improvement has been seen in the 5-year survival rate [3] of OSCC in the last 50 years [4]. The reason for this may be the late stage diagnosis and no early detection of a reliable diagnostic marker. The high morbidity and mortality rate in OSCC patients [5] ultimately led to the aspiration for enhanced knowledge of the characteristics and pathogenesis involved, as clinical and histological analyses are the only basis for OSCC diagnosis, furthermore, specific biomarkers are highly supportive of untraceable hidden sites of OSCC and for the screening of high risk patients [4]. Since OSCC is a multifactorial disease, molecular pathways are necessary for diagnostics, prognostics and treatment of cancer [2], for which the human genome database (HGD), the proteomic approach, introduced by novel technology, is developed to monitor genetic alterations and to recognize protein biomarkers linked with growth, progression and recurrence of the tumour [6]. In comparison to tissue biopsy which is invasive, there have been plenty of biomarkers identified, amongst them, the most recommended biomarker detection medium for OSCC includes biomarkers in body fluids, such as blood and saliva (serum and plasma) [7]. Due to the surplus pool of biomarkers available for OSCC detection, saliva reflects an advantageous medium because of its easy accessibility; less complexity than blood; and inhibitory substances, with it being an accurate, cost-effective and non-invasive technique [8]. The objective of this review is to discover potential specific biomarkers and their utility for oral cancer detection.

**Figure 1:**
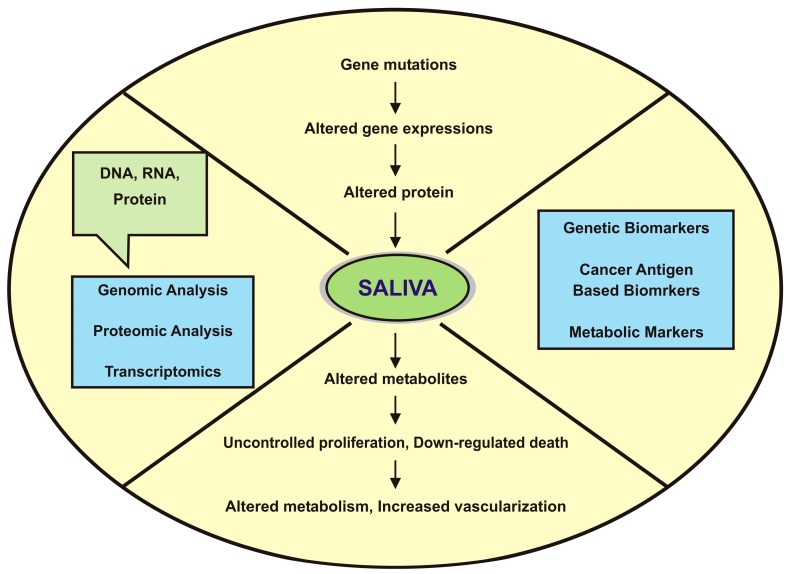
Clinical utility of saliva and the process showing carcinogenesis prospects for biomarkers.

Cancer cell transformation and progression involves complex events, including upregulation and downregulation of a variety of genes which are essential for cell proliferation, differentiation and cell death. Consequently, an analysis of proteins gives an accurate prediction of the function of marker proteins (see Figure 1) [4]. Hence, the transformation of oral mucosal epithelial cells to malignant OSCC is surrounded by saliva, and besides that, salivary secretions are mixtures of complex proteins, carbohydrates, lipids, electrolytes and water. The combination of serous and mucinous mixed secretions is known as whole mouth saliva (WMS) [9], which consists of several proteins, gingival crevicular fluid (GCF), cellular debris, microorganisms and serum components required to detect salivary protein markers for OSCC detection [10].

## 2. Proteomic Tools and Saliva Sampling

According to the types of biomarkers, potential salivary biomarkers are investigated in the following ways: high performance liquid chromatography (HPLC), enzyme-linked immunosorbent assay (ELISA), radioimmunoassay, two-dimensional gel electrophoresis (2DE), followed by mass spectrometry (MS), 2DE and reverse-phase liquid chromatography (LC), followed by LC-tandem MS, matrix-assisted laser desorption/ionization time-off light mass spectrometry (MALDI-TOF MS), and 2DE followed by MALDI-TOF MS.

After collection of the saliva sample, a centrifugation process is frequently performed. Whole saliva in an unstimulated state contains solid constituents such as keratin debris, blood cells, desquamated oral epithelial cells, and bacteria, therefore after separation of solid constituents, samples are stored in a frozen state until analysis and until the supernatant (cell free) portion of the saliva sample has been discarded and the pellet portion of the saliva has been used. The pellet portion is washed by supernatant after centrifugation of whole saliva, and is most often used for diagnostic purposes [11]; cell lysis proteins were extracted from the pellet portion and subjected to trypsin for digestion which results in a complex peptide mixture [12]. In the search for cancer biomarkers, standardized multiple salivary collection methods with minimum inconsistency and circadian fluctuations must be used for proteomic screening [13]. Hence, saliva sample collection is performed by two methods, known as stimulated whole saliva collection and unstimulated whole saliva collection. One of the ways to stimulate saliva is to chew a standardized bolus i.e., paraffin or a gum base, until it becomes soft, to generate a reflex masticatory response or the use of a stimulant i.e., citric acid [10] which yields a greater volume of saliva production as compared to stimulated saliva. This can be collected simply by spitting, suction, swab, drain or drooling; passive drooling is a preferable method due to its proximity between salivary glands (locally) and serum (systemically) that renders whole saliva an attractive fluid for biomarker studies [14].

**Figure 2:**
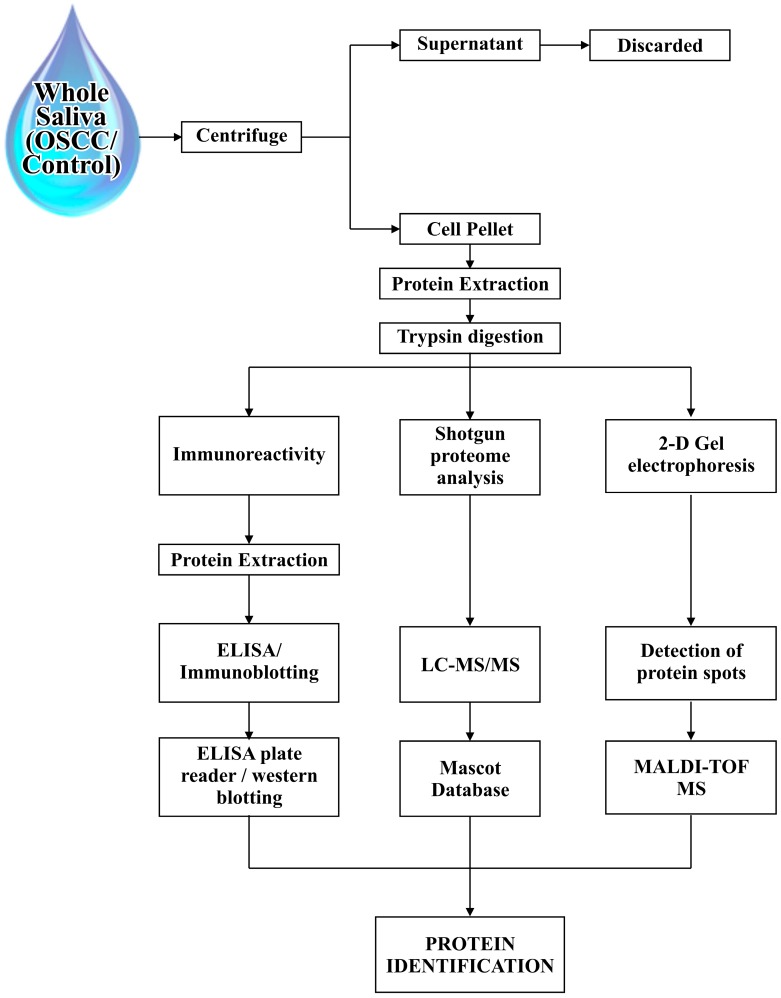
Schematic flow of the steps used for the biomarkers extraction from human saliva of Oral squamous cell carcinoma (OSCC) patients and healthy individuals.

The next step is to determine proteins in supernatants, for which different methods can be used, such as an immune-reactivity assay for salivary markers, shotgun proteome analysis, and two-dimensional gel electrophoresis; the immune-reactivity assay includes ELISA kits. Furthermore, immune-blotting can be done for comparisons of protein analysis; two-dimensional gel electrophoresis is used for the detection and matching of protein spots between the diseased and control samples, which is further analysed by mass spectrometry by a variety of approaches, using matrix-assisted laser desorption ionization with time-of-flight mass spectrometry (MALDI-TOF MS), and MALDI tandem MS, using MALDI–TOFTOF mass spectrometry. The shotgun proteome analysis is based on reversed phase liquid chromatography (RP-LC) and the subsequent LC-tandem mass spectrometry (LC-MS/MS) analysis, which can be done by the LC packing nano-LC system with a nano-electrospray interface and QqTOF mass spectrometer, and hence identification of the peptides and proteins was analysed by using the Mascot database search engine (see Figure 2).

## 3. Potential Salivary Biomarkers for Oral Cancer Detection

Saliva is preprogramed to respond to certain events in an oral cavity, therefore the first biomarker for breast cancer is HER2/neu, found in saliva [15]. In view of this fact, tumour progression and metastasis show a difference in protein expression levels which is advantageous to monitor patients at cancer risk [16]. Katakura et al, in 2007, examined 20 healthy patients and 19 patients with oral cancer and checked levels of cytokines (IL-6, IL-8, IL-β1) and osteopontin in saliva through ELISA, which showed higher levels of cytokines in oral cancer patients as compared to the control group; IL-6 levels showed significant elevation in oral cancer patients while this was not detected in the control group. However, IL-8 and IL-β1 levels were found in both groups with definite increased levels in oral cancer patients, which therefore suggested saliva as an important screening tool for oral cancer. Head and neck cancer salivary proteome is different than normal saliva [13]. Hence, identification of proteins is either fractionated by gel electrophoresis or digestion by enzymatic procedures to produce peptide mixtures [17]. So far, 3000 proteins have been identified in saliva by using various analytical platforms, advances in mass spectrometry and a combination of data from multiple groups [18]. A strong board of candidates with high specificity and sensitivity is required for the detection of OSCC amongst them; high levels of CD44 show a strong association to differentiate malignant from benign lesions [19] shown in Table 1. Correspondingly, in the saliva of OSCC patients, three known markers were found to be four-fold increased, such as cytokeratin 19 fragment (Cyfra21–1), cancer antigen 125 (CA-125) and tissue polypeptide antigen (TPS) [20]; fibrin; transferrin; Ig-heavy chain constant region; cofilin-1 [21]; salivary endothelial levels [22]; pro-inflammatory cytokine interleukin-6 (IL-6); TNF-a; and antibodies responsive to gene aberrations, such as anti-p53 antibodies, were also identified [23]. The latest techniques have been followed by Gallo et al, in 2016 [24], for the identification of OSCC proteomic signatures; Gallo et al created a predictive model and analysed it through SELDI-TOF mass spectrometry of saliva from 45 OSCC patients and 30 control patients to investigate the diagnostic and prognostic potential of proteins in saliva. Different neural networks were used, i.e., feed-forward (FF), which showed prognostic accuracy; and radial basis function (RBF), which showed diagnostic accuracy which clearly indicated that the struggle of selecting a particular predictive model and the research related to it remains under investigation. These potential biomarkers identified from the saliva of OSCC patients are listed in Table 1.

Receiver operating characteristic analysis is required to evaluate the diagnostic value of discovered candidate biomarkers for OSCC, i.e., the value of sensitivity and specificity [15]. Recent advancements showed that a population based study was reported recently to measure the tobacco specific nitrosamine (TSNAs) carcinogen from oral fluid and concluded that *N′*-nitrosonornicotine NNN is associated with oesophageal and oral cavity cancers [49]. A set of panels were statistically analysed by Proceedings of the National Academy of Sciences of the United States of America (PNAS), containing four proteins—MMP1, KNG1, ANA2, and HSPA5—which appeared to be suitable for detecting OSCC cases in Taiwan’s Oral Cancer Screening Program published by Medical News Bulletin under the section of Detecting Oral Cavity Cancer with a Saliva Sample on 16 October 2016 [48].

## 4. Conclusions

In the field of oral cancer research, salivary proteomics is rapidly advancing and evolving by the identification and use of novel biomarkers for early detection and prognostication approaches, including metabolic, proteomics, genomics and bioinformatics. The systemic analysis of salivary proteomics biomarkers and screening of saliva offers an attractive diagnostic tool to turn salivary diagnostics into clinical and commercial reality to combat oral cancer. Due to the cellular and molecular heterogeneity of OSCC progression, multiple genes are potentially involved in oral carcinogenesis, hence a panel of several potential biomarkers can make a precise diagnosis rather than any marker alone. Furthermore, due to the easily obtained characteristics of saliva, such as its proximity to the oral cavity and non-invasive collection procedure, the focus on OSCC has and will be more shifted to serum and saliva analysis instead of tissue analysis. Therefore, experience gained in OSCC salivary biomarkers serves as an essential reference for cancer detection and for monitoring non-cancerous disease activity. Salivary diagnostics with the development of novel technologies—mass spectrometry, gel electrophoresis, chromatography, microarrays, high performance liquid chromatography (HPLC), polymerase chain reaction (PCR) and enzyme-linked immunosorbent assay (ELISA)—is a flourishing field associated with point-of-care technologies, electrochemical detection, RNA sequencing, and liquid biopsy. Furthermore, primary screening through saliva could be the best choice with the advancements in proteomics and genomics. The extensive discovery of novel biomarkers and their validation will transform the field of diagnosis for oral cancers and even for non-cancerous activities. This update gives a comprehensive review of the emerging diagnostic proteomic tools and biomarkers for the early detection and diagnosis of OSCC through saliva.

## Figures and Tables

**Table 1:** Biomarkers of saliva for cancer diagnosis.

Identified Salivary Biomarkers	Proteomic Tools	Inference	References
Glutathione	HPLC	Epidemiological marker to identify subjects with an increased risk of developing oral squamous cell carcinoma (OSCC) to submit strict follow up and chemoprevention.	[25]
Interleukin 1a (IL-1a), Interleukin 1b (IL-1b), Interleukin-6(IL-6), Interleukin-8(IL-8), TNF-a	ELISA	These pro-angiogenic, pro-inflammatory cytokines were found to be elevated in the whole saliva of oral cancer patients and oral pre-cancers as compared to controls, which suggested its utility as surrogate indicators of carcinogenic transformation from oral pre-cancer to oral cancer.	[26,27,28]
CD44	Immunoblot	CD44 is elevated in the majority of head and neck squamous cell carcinoma (HNSCC) and distinguishes cancer from benign diseases with high specificity; these markers will detect HNSCC with very high sensitivity and specificity.	[19]
CD59	Immunoblot	Non-invasive method for the diagnosis of oral cancer.	[15]
Immunoglobulin heavy chain constant region gamma (IgG)	LC/MS	Significantly altered in OSCC patients as compared with healthy controls; they are inhibitors of apoptosis.	[21]
Mac-2 binding protein (M2BP)	ELISA	Provide a sensitivity of 90% and a specificity of 83% for OSCC detection.	[15]
MRP14	Immunoblotting	MRP14 is a calcium-binding protein that has been implicated in different types of human cancers. Provides a sensitivity of 90% and a specificity of 83% for OSCC detection.	[15]
p53 antibodies	ELISA	Presence of p53 autoantibodies in saliva, as well as serum of oral cancer patients demonstrated that its detection in saliva can offer a non-invasive method for the detection of a subset of tumors with p53 aberrations.	[29,30]
Profilin	Immunoblot	The data proved that these new targets may lead to a simple clinical tool for the non-invasive diagnosis of oral cancer and suggested that patient-based salivary proteomics is a promising approach to the discovery of biomarkers for oral cancer detection.	[15]
S100 calcium binding protein	LC/MS	S100A2, an 11.4 kDa protein, is a member of the S100 family of calcium-binding proteins that have diverse functions, regulating a variety of cellular processes such as differentiation, regeneration, cell growth, and signal transduction in neoplastic cells and is a prognostic marker for oral cancer patients.	[21,31]
Endothelin-1	Quantitative real time RT-PCR	Salivary ET-1is a good biomarker for OSCC development in oral lichen planus (OLP) patients regardless of the degree of OLP disease activity. However, it appeared not to be a good biomarker for detecting recurrence of OSCC in patients in remission.	[22,32]
Cofilin-1	LC/MS	These proteins are involved in tumour progression, metastasis and angiogenesis.	[21]
Albumin	MALDI-MS	Serum albumin levels decreased in oral pre-malignancy and oral malignancy cases compared to healthy individuals. Salivary albumin levels increased in oral pre-malignancy and oral malignancy cases compared to healthy individuals, suggesting that albumin may play a role in the early diagnosis and prognosis of oral pre-malignant and oral malignant tissues.	[33,34]
Telomerase	PCR and ELISA	Telomerase is required for the maintenance of telomere length during chromosome replication; telomerase activity has been detected in tumor cells.	[35]
Tissue polypeptide antigen (TPA), Cyfra 21-1, Cancer antigen 125 (CA-125)	ELISA, TRFIA, Immuno-radiometric assay	Significant increases in salivary concentrations of Cyfra 21-1, CA-125 and tissue polypeptide antigen markers revealed sensitivity, specificity, and it is used as a diagnostic tool, especially when a concurrent analysis for significantly increased markers is done.	[20,36,37]
Transferrin	LC/MS	Salivary transferrin levels in patients are strongly correlated with the size and stage of the tumor.	[21,38]
Fibrin	LC/MS	Similarly, the use of the fibrin SCC biomarker is limited by its non-specificity, even though it is involved with various carcinogenic processes.	[21,39]
α-Amylase	MALDI-MS	α-amylase (57 kDa) dominated the high mass range in the MALDI mass spectra of the saliva from healthy subjects, but the peak was suppressed for patients with oral cancer. SDS-PAGE results show that concentrations of alpha-amylase in patients' saliva were significantly higher than those in healthy subjects. MALDI-MS thus has potential as a possible rapid diagnostic screening tool for oral cancer.	[40]
Salivary zinc finger, Protein 510 peptide	MALDI-TOF MS Technology	ZNF510 peptides, as OSCC-related salivary biomarkers via the proteomic approach, proved useful in adjunct diagnosis for early detection rather than as a specific diagnosis marker for progression of OSCC patients.	[41]
Keratin 36, cystatin A.	MS-based proteomics	Keratin overexpression in OSCC cells may have important molecular functions as structural constituents of the cytoskeleton as well as implications on cell shape and cell size. A 14 kDa protein detected in pre-treatment saliva from the OSCC patients was identified as a truncated cystatin SA-I, with deletion of three amino acids from the N-terminus, proposing that Protein-Chip analysis may provide a reliable screening test and cystatin SA-I might be a useful tumor biomarker for OSCC.	[42,43]
Truncated cystatin SA-I	Anion exchange (Q10), cation-exchange (CM10), reversed phase (H50), and immobilized affinity capture (IMAC3) Protein-Chip array	A 14 kDa protein detected in pre-treatment saliva from the OSCC patients was identified as a truncated cystatin SA-I, with deletion of three amino acids from the N-terminus. Truncated cystatin SA-I is a useful tumor biomarker for OSCC.	[43]
Myosin, actin, S100A7, keratin-19 and catalase	iTRAQ labeling and Mass spectrometric analysis, Immunoblot	Actin and myosin are promising salivary biomarkers for distinguishing premalignant and malignant oral lesions. It is highly beneficial and noninvasive, being an effective alternative to serum testing, and it provides the possibility of developing self-, home-testing kits for such markers, further facilitating it as a diagnostic aid.	[44,45]
Signal transducer and activator of transcription 3(STAT3), Serpin B3 (SCCA1)	(1) preparative IEF using free flow electrophoresis (FFE), (2) SCX chromatography, and (3) LC on line with ESI-MS/MS	Transcription factor that binds to the interleukin-6-responsive elements, may act as a protease inhibitor to modulate the host immune response against tumor cells.	[12]
α-1-antitrypsin (AAT), haptoglobin (HAP)	2DE and MS	The patients' saliva α1-antitrypsin (AAT) and haptoglobin (HAP) β-chains were resolved into polypeptide spots with increased micro heterogeneity. A strong association of AAT and HAP with OSCC was further supported by immunohistochemical staining of cancer tissues.	[46]
Thioredoxin	MALDI–MS and LC-MS/MS	Saliva thioredoxin mRNA level was concordantly up-regulated in OSCC subjects. In addition, thioredoxin was found over-expressed in human cancers such as non-small cell lung, gastric, cervical and hepatocellular carcinomas.	[47]
KNG1, ANA2, and HSPA5	Multiple reaction monitoring-MS	Four-protein panel offers a clinically effective tool for detecting OSCC and monitoring high-risk oral premalignant diseases (OPMDs).	[48]
Tobacco specific nitrosamines (TSNAs), N'-nitrosonornicotine (NNN)	A simple method with an alkaline single liquid–liquid extraction with dichloromethane/isopropanol was used for quantification.	Biomarker of cancer risk associated with exposure to tobacco smoke.	[49]
AAT and HAP	Two-Dimensional Electrophoresis, Mass spectrometry	Panel of proteins is useful for the prediction of aggressive phenotypes in OSCC; and the distinctive expression of proteins and tumor size parameter shows the aggression of cancer.	[50]
Secretory leukocyte peptidase inhibitor, keratin 36, cystatin A.	Mass spectrometry	Non-invasive biomarker of oral cancer progression with potential in preventive treatment.	[51]

High performance liquid chromatography (HPLC), enzyme-linked immunosorbent assay (ELISA), liquid chromatograph/mass spectrometer (LC/MS), matrix-assisted laser desorption/ionization mass spectrometry (MALDI-MS), polymerase chain reaction (PCR), time-resolved fluoro-immunoassay (TRFIA).
